# Integrated assessment of short-term direct and indirect economic flood impacts including uncertainty quantification

**DOI:** 10.1371/journal.pone.0212932

**Published:** 2019-04-04

**Authors:** Tobias Sieg, Thomas Schinko, Kristin Vogel, Reinhard Mechler, Bruno Merz, Heidi Kreibich

**Affiliations:** 1 GFZ German Research Centre for Geosciences, Section Hydrology, Telegrafenberg, Potsdam, Germany; 2 University of Potsdam, Institute of Environmental Science and Geography, Karl-Liebknecht-Strasse 24-25, Potsdam, Germany; 3 International Institute for Applied Systems Analysis (IIASA), Risk and Resilience (RISK) Program, Schlossplatz 1, Laxenburg, Austria; Bristol University/Remote Sensing Solutions Inc., UNITED STATES

## Abstract

Understanding and quantifying total economic impacts of flood events is essential for flood risk management and adaptation planning. Yet, detailed estimations of joint direct and indirect flood-induced economic impacts are rare. In this study an innovative modeling procedure for the joint assessment of short-term direct and indirect economic flood impacts is introduced. The procedure is applied to 19 economic sectors in eight federal states of Germany after the flood events in 2013. The assessment of the direct economic impacts is object-based and considers uncertainties associated with the hazard, the exposed objects and their vulnerability. The direct economic impacts are then coupled to a supply-side Input-Output-Model to estimate the indirect economic impacts. The procedure provides distributions of direct and indirect economic impacts which capture the associated uncertainties. The distributions of the direct economic impacts in the federal states are plausible when compared to reported values. The ratio between indirect and direct economic impacts shows that the sectors Manufacturing, Financial and Insurance activities suffered the most from indirect economic impacts. These ratios also indicate that indirect economic impacts can be almost as high as direct economic impacts. They differ strongly between the economic sectors indicating that the application of a single factor as a proxy for the indirect impacts of all economic sectors is not appropriate.

## Introduction

Flood events can have multiple impacts on economic sectors at all scales of an affected region. Those are not limited to direct impacts on companies, which commonly occur inside the flooded areas, but also include indirect impacts across economic sectors, which typically occur outside the flooded regions [1, 2], by e.g. affecting other companies due to disruptions in the production chain. Comprehensive impact assessments that capture both, direct and indirect economic impacts, are needed to inform flood risk management [3, 4] and are increasingly demanded by decision makers [5, 6]. Relying on cost-benefit analyses that exclude indirect economic impacts may lead to sup-optimal decisions [7, 8]. Yet, integrated assessments of direct and indirect economic impacts on companies and economic sectors are rarely conducted [9].

The most common approach to estimate direct flood damage to buildings goes back to Grigg & Hellweg [10], who related the water level at the buildings to the damage caused by the flood. These so-called depth-damage curves consider the water level as the only parameter and are the base of many flood damage models [11–14]. Recently, multi-variable models consider additional parameters, e.g. precautionary measures, building type or contamination, to improve the description of the damage processes [15–19]. However, most of these approaches estimate the damage to private households and only a few methods aim to predict flood damage suffered by companies [20]. Recent advances introduced tree-based models for the estimation of flood damage to different company assets, i.e. buildings, equipment, and goods and stock [21]. It has also been shown that a sector-specific consideration of flood damage increases the model performance, suggesting that direct economic impacts should be assessed separately for different economic sectors [21].

The performance of models estimating the direct economic flood impacts to companies is generally low [21, 22], and uncertainty assessments are needed for evaluating the model reliability. Yet, uncertainties associated with the estimation of direct economic impacts are rarely quantified [2]. A recent study proposes the use of multi-model ensembles to combine estimations from different models of the same class aiming to capture the uncertainties [23]. However, this covers only the uncertainties associated with the model setup and the parameter estimation, while other components such as the water level or the exposure data also contribute to the overall uncertainties [24].

Another source for inaccurate estimates of direct economic impacts is the inconsistent handling of spatial scales. Direct economic impacts can be estimated at different spatial scales [25], which can be divided into the object, regional and national level. Most models are derived at the object level, but applied at the regional level using different kinds of exposure data, e.g. land use data sets at the regional level versus building data sets at the local level. This leads to inconsistent methodologies across scales [26]. In addition, these land use data sets suffer from inconsistencies themselves, such as varying density of objects within an area, which is not detected by the land use data [27].

Yet, the assessment of direct economic flood impacts on the national scale is still mostly conducted on the basis of land use [20]. One example for the use of individual objects on the regional scales is the study of Huttenlau et al. [28] who assessed direct economic flood impacts in Tyrol, Austria. However, the uncertainties associated with the estimations are not considered. Saint-Geours et al. [29] attempt to assess uncertainties e.g. associated with depth-damage curves and the hazard maps in a probabilistic framework, while using a land use data set instead of single objects for the exposure. The method used in this study combines the object-based approach with a probabilistic attempt to take all uncertainties associated with the estimation of direct economic impacts into account and represent them within the results [26]. This approach can be applied independent of spatial scales making the estimation seamless.

Indirect economic impacts of natural hazards are mostly assessed with macroeconomic models that describe interactions between different economic sectors as well as the public sector, e.g. Input-Output (IO) models and Computable General Equilibrium (CGE) models [5]. IO and CGE models are usually applied at the regional or national scale. For example, Santos & Haimes [30] developed a so-called inoperability model based on the IO analysis to estimate the production loss of an economic system resulting from unexpected events. Rose & Liao [31] applied a CGE model to assess the resilience of regional economies to disasters.

These macroeconomic modeling techniques have also been used for assessing flood impacts. For example, Hallegatte [32] developed an adaptive regional IO model to estimate the indirect economic impacts caused by Hurricane Katrina. Mochizuki et al. [33] estimated the follow-on impacts of a flood in Cambodia with an IO based inter-industry economic model, and Carrera et al. [34] used a CGE model [35] to assess indirect economic impacts caused by a flood event of the Po river in Italy.

However, most flood impact assessments neglect indirect economic impacts [2, 36]. Some models, as e.g. the Damagescanner, include indirect effects simply as a certain percentage of the direct economic impact [37]. Furthermore, IO and CGE models incorporate damage estimates from direct impact assessments only as exogenous point-estimated parameters. Only few studies aim to directly link the estimation of direct economic impacts with the assessment of indirect economic impacts. The existing approaches mainly use land use-based single parameter models, as the depth-damage curves, to estimate the direct damage caused to a system. Thus, Koks et al. [9] and Jonkman et al. [1] couple depth-damage curves with IO based models, whereas Carrera et al. [34] link depth-damage functions with CGE models. These approaches, however, largely ignore uncertainties associated with the estimation of direct economic impacts, since they typically only perform a sensitivity analysis over a rather limited number of exogenous direct damage estimates. Moreover, these models are not able to capture spatial inconsistencies, which typically occur in the exposure data sets such as land use data [25, 38]. So far, a coupling procedure which quantifies and finally forwards the uncertainties associated with the estimation of the direct economic impacts to the estimation of the indirect economic impacts within one methodological approach is missing.

Therefore, this study develops a novel procedure for a comprehensive quantitative assessment of economic flood impacts that (1) includes uncertainties associated with the estimation of direct impact [26] and (2) subsequently links these impacts to a macroeconomic IO-based assessment of indirect economic impacts. In the case of extreme event risk, the short-term indirect economic spillover effects caused by supply-side shocks are of particular interest. Hence, a supply-side IO model is chosen, since it is more applicable to capture short-term economic impacts than e.g. a CGE model [39]. Short-run substitution and price elasticities, as used in the CGE models, are close to zero for short-term assessments [40] making CGE models more complicated to apply for the estimation of short-term impacts [41].

The study focuses on two main aspects. The first aspect is the application of seamless estimation and uncertainty quantification of direct economic flood impacts to the national level by means of newly available exposure data sets. The second aspect is the linkage between the estimation of the direct and indirect economic flood impacts on a national level, including the uncertainties associated with the estimation of the direct economic impacts.

## Data and methods

**Fig 1:**
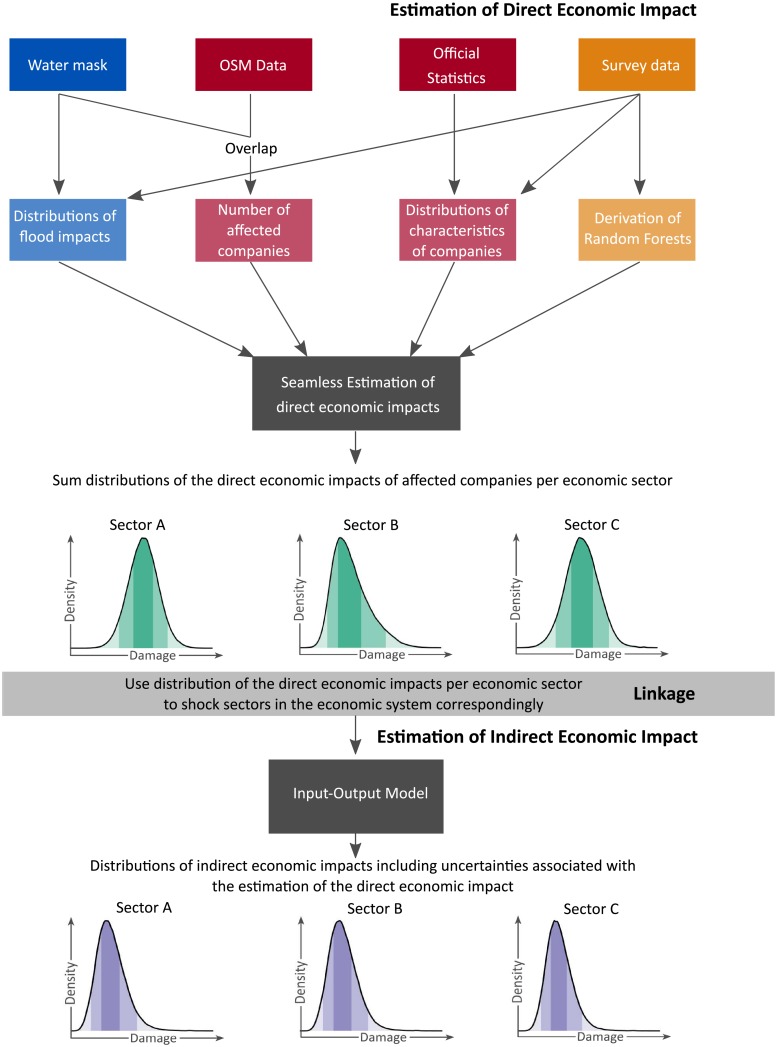
Modeling procedure and data sets (darker colours) used to derive inputs (brighter colours) for the hazard (blue), exposure (red) and vulnerability (orange) used by the method for the computation of the direct (green) and indirect (purple) economic impacts.

Fig 1 shows the procedure developed in this study and the data sets used. The procedure is applied to the flood event in 2013 in Germany, which caused severe impacts and affected large parts of the country along the rivers Elbe and Danube for several weeks [42–44]. In the following, the estimation of the direct economic effects is described, followed by the presentation of the estimation of the indirect effects and of the linkage between direct and indirect damages. R (version 3.4.1)—A language and environment for statistical computing [45] is used for all analyses and figures and for the implementation of the whole procedure.

### Direct economic effects

Direct economic impacts are estimated by the probabilistic, object-based approach developed by [26]. Tree-based damage models [21] are used to compute the relative damage to single companies. To transfer relative to absolute damage the net fixed assets per company are estimated. The first row in Fig 1 shows the used data sets and how they contribute to the prediction of direct economic impact.

#### Data sets

Twelve federal states were affected during the flood event in 2013. For this study the eight federal states along the catchments of the rivers Danube and Elbe are considered, which were most affected by extensive flooding [43, 44]. This comprises the federal states Bavaria, Brandenburg, Lower Saxony, Mecklenburg, Saxony, Saxony-Anhalt, Schleswig-Holstein and Thuringia, which suffered more than 98% of the overall direct economic impacts in Germany [44].

The flooded area and the related water depths are taken from a water mask provided by JBA Risk Management (http://www.jbarisk.com). A data base of single buildings in Germany is extracted from openstreetmap.org (OSM). Only buildings with an occupancy related to a commercial, educational, industrial, public or mixed use are counted for the number of affected companies in the federal states. Buildings related to infrastructure, government or gathering venues are excluded for the counting.

Official statistics, taken from the German Federal Statistical Office, are used to derive the characteristics (size, economic sector, net fixed assets) of companies in different federal states. The data sets can be accessed via the GENESIS online data base (https://www-genesis.destatis.de/genesis/online/). Data regarding the distribution of company size classes (number of employees grouped in classes) and economic sectors separated by different federal states are taken from the German Federal Statistical Office, GENESIS Table no. 52111-0003. Net fixed assets of buildings and equipment from different economic sectors are taken from the national accounts (VGR des Bundes), GENESIS Table no. 81000-0117. Table 1 shows the net fixed assets of buildings and equipment and the number of employees of different economic sectors. Table 2 shows the distribution of companies among the different economic sectors and size classes. The economic sectors comprise private companies e.g. from the manufacturing or commercial sector as well as public administrations as e.g. education or arts.

**Table 1:** Economic sectors and sector groups, net fixed assets (buildings and equipment) in millions of Euros and the size in thousands of employees for different economic sectors throughout all of Germany. Different damage models are applied to the four sector groups.

Sector group	Economic sector	buildings	equipment	size
*Manufacturing*				
	Manufacturing	131000	335000	7442
	Electricity, gas,			
	steam and air conditioning supply	130000	46484	256
	Water supply; sewerage;			
	waste management and remediation activities	266000	19609	261
	Construction	22469	24132	2426
*Commercial*				
	Wholesale and retail trade			
	repair of motor vehicles and motorcycles	134000	638000	5903
	Transporting and storage	260000	144000	2084
	Accommodation and food service activities	36600	12400	1774
*Financial*				
	Information and communication	37197	45259	1218
	Financial and insurance activities	129000	10426	1194
	Professional, scientific and technical activities	61343	30716	2577
	Administrative and support service activities	38016	225000	2960
*Service*				
	Education	253000	18024	2369
	Human health and social work activities	333000	79094	5195
	Arts, entertainment and recreation	14699	11909	644
	Other services activities	44323	13843	1488

**Table 2:** Percentages of companies in different size classes and economic sectors in the eight federal states.

Sectorname	1-10	11-50	51-250	>250	total
Manufacturing	5,506	1,370	0,471	0,112	7,46
Electricity, gas, steam and air conditioning supply	2,490	0,026	0,016	0,005	2,54
Water supply; sewerage; waste management and remediation activities	0,301	0,090	0,030	0,003	0,42
Construction	12,260	1,14	0,103	0,008	13,51
Wholesale and retail trade repair of motor vehicles and motorcycles	17,900	1,560	0,245	0,039	19,74
Transporting and storage	3,068	0,486	0,095	0,013	3,66
Accommodation and food service activities	6,985	0,468	0,052	0,004	7,51
Information and communication	3,071	0,223	0,056	0,009	3,36
Financial and insurance activities	2,003	0,045	0,036	0,020	2,10
Professional, scientific and technical activities	13,048	0,661	0,080	0,013	13,80
Administrative and support service activities	5,628	0,408	0,143	0,032	6,21
Education	1,844	0,380	0,059	0,010	2,29
Human health and social work activities	6,137	0,860	0,239	0,065	7,30
Arts, entertainment and recreation	2,677	0,093	0,016	0,003	2,79
Other services activities	6,938	0,288	0,051	0,008	7,29

A survey data set, collected in the aftermath of flood events in Germany, is used to derive models predicting direct economic flood impacts and to derive distributions used for the seamless estimation (Table 3). Computer Aided Telephone Interviews were carried out approximately a year after the events in 2002 and 2013 respectively. Interviewed companies were chosen from a site-specific random sample based on lists of affected streets in the corresponding areas and the person with the best knowledge about the flood damage was questioned for each company [46]. The answers given were cross-checked during the interview to clarify contradictory statements and to ensure data quality. See Kreibich et al. [46, 47] for further details about the survey and the data processing as well as Sieg et al. [21] for the development of the tree-based models and a basic statistical analysis of the data sets.

**Table 3:** Input variables used by the Random Forests for the estimation of the direct economic impacts and the distributions used to simulate these variables as well as data sources.

	Input Variable	Data Source	Distribution
*Flood characteristics*			
	Water level	Water mask	Gamma
	Inundation Duration	Survey	Gamma
	Contamination	Survey	Multinomial
*Company characteristics*			
	Mitigation	Survey	Multinomial
	Adaptation	Survey	Multinomial
	Emergency actions	Survey	Multinomial
	Size	Survey	Gamma
	Size class	Official statistics	Multinomial
	Sector	Official statistics	Multinomial
	Spatial situation	Survey	Multinomial

#### Estimation of direct economic impacts

Direct economic impacts are estimated at the object level. They are determined by hazard, exposure and vulnerability characteristics. Here, the hazard is represented by the water level, inundation duration and contamination at the companies during the flood event in 2013 in Germany. The exposure consists of the number of affected companies, the characteristics of the companies (size, economic sector, precaution etc.) and the asset values of the companies. The vulnerability is described by a tree-based damage model.

Since the individual characteristics of the affected companies are unknown, the broad range of individual specifications was reflected by sampling company characteristics from distributions derived by a water mask, official statistics and survey data. Table 3 shows the input variables (predictors) used for the damage modeling as well as the data sources and distributions, which are used to sample the predictors. The parameters of the distributions can be obtained from Sieg et al. [26]. The flood impact at each company is sampled from distributions derived from the water mask and survey data, whereby the water level is taken from the water mask and the inundation duration as well as the contamination are taken from the survey data.

The number of directly affected companies in each federal state is determined by an overlap of the water mask and the OSM data. The affiliation of single companies to specific economic sectors is sampled from official statistics. Company characteristics, such as precautionary measures or the size of a company, are sampled from official statistics and survey data. The net fixed assets of a company are calculated by multiplying the number of employees with the net fixed asset value per employee of the corresponding economic sector (Table 1). It is assumed that the net fixed assets of buildings and equipment per employee do not vary strongly within Germany. However, to account for small variations net fixed asset values are varied uniformly by ± 10%. 1000 versions of affected company sets are sampled for each federal state to simulate the distribution of the input variables. This procedure is in contrast to state-of-the-art models using land use data, which typically use the mean value of the input variables and thus ignore the variability of the exposed assets.

For each of the sampled company version the relative damage is estimated with a tree-based model, that is derived from the survey data collected after the 2002 and 2013 flood events [21]. Individual damage models are used for the four sector groups (Table 1), that are formed according to the European statistical classification of economic activities in the European Community (NACE Rev. 2; Nomenclature statistique des Activites economiques dans la Communaute Europenne) [48]. In total, eight different Random Forests are grown—following the algorithm of [49]—to consider the four different economic sector groups (1) manufacturing, (2) commercial, (3) financial and (4) service, as well as two different types of assets (a) buildings and (b) equipment. We refer to [21] for a detailed description of the application of the Random Forest approach for the estimation of direct economic flood impacts to companies. For a general introduction to Random Forest we refer to [50].

Previous studies on tree-based flood damage models usually take the mean value of observations with comparable input variables as an estimator for the relative damage [16, 21, 51–53]. They consequently ignore the large variation of damage realizations, that are typically observed for comparable input variables. Here we aim to capture the uncertainty related to the prediction of the direct economic impacts and follow the algorithm of [54] to obtain a distribution of direct economic impacts instead of a point estimate.

The probability distributions for the relative damage of a single company is transferred to absolute values by multiplying the relative damage with the net fixed asset values. To obtain the direct economic impact per federal state, the absolute values of the damage distributions are summed up. Similarly, the direct economic impacts to individual economic sectors can be estimated by summing up absolute economic impact estimates for the affected companies in the corresponding sector. The latter distributions are used to shock the IO model, as illustrated in Fig 1.

### Indirect economic impacts

A national IO table of the economy of Germany for the year 2013 was obtained from the German Federal Statistical Office. On this basis a supply-side IO model was used to compute the indirect economic effects.

#### Input-Output tables

The IO table, taken from the German Federal Statistical Office, can be accessed via the GENESIS online data base with the table no. 81511-0003. It consists of 72 economic sectors. For this study the table was aggregated to 19 economic sectors following NACE Rev. 2 according to the European statistical classification of economic activities in the European Community [48]: Agroforestry; Mining and Quarrying; Manufacturing; Electricity, Gas, Steam, and Air Conditioning Supply; Water Supply, Sewerage, Waste Management and Remediation Activities; Construction; Wholesale and Retail Trade, Repair of Motor Vehicles and Motorcycles; Transportation and Storage; Accommodation and Food Service Activities; Information and Communication; Financial and Insurance Activities; Real Estate Activities; Professional, Scientific and Technical Activities; Other Economic Activities; Administrative and Support Service Activities; Education; Human Health and Social Work Activities; Arts, Entertainment and Recreation; other Service Activities.

#### Input-Output models

IO models originate from the work on economic problems of Wassily Leontief [55]. The core of an IO model is observed data which reflects economic activities between different producing sectors. Table 4 shows a schematic IO table, consisting of the intermediate flows **Z** of values/products between the sectors, which are producing and purchasing the products to each other. Traditionally, Input-Output models can be used to estimate the changes of the inputs *x*_*j*_ resulting from a change in the outputs *x*_*i*_, e.g. final demand *f*. This can be estimated as follows:

$$\begin{array}{c} x={\left(I-A\right)}^{-1}f \end{array}$$ (1)

with $A=Z{\hat{x}}^{-1}$ and $\hat{x}$ as the diagonal matrix of *x*, whereby (*I* − *A*)^−1^ is known as the so-called Leontief inverse. This model is often referred to as demand-side IO model.

**Table 4:** Schematic Input-Output table with Z denoting the intermediate flow matrix, the final demand *f*, the capital stock *K*, labour *L* and imports *m*.

		Purchasing Sectors						
		1	…	*j*	…	*n*	Final Demand	Total Output (x)
Producing Sectors	1							
	…							
	*i*			**Z**			*f*	*x*_*i*_
	…							
	*n*							
Payments Sector	Value added (**v**′)			*K*_*j*_				*K*
				*L*_*j*_				*L*
	Imports			*m*_*j*_				*M*
Total outlays (**x**′)				*x*_*j*_				*X*

[56] introduced the alternative IO model, which can be used to estimate the change of the outputs *x*_*i*_ resulting from a change of the inputs *x*_*j*_, e.g. the capital stock *K*_*j*_ by changing the “column-wise” view of the demand-side model to a “row-wise” view.

$$\begin{array}{c} x={\left(I-B\right)}^{-1}v \end{array}$$ (2)

with $B={\hat{x}}^{-1}Z$.

For a comprehensive introduction of demand-side and supply-side IO models see [57].

Originally, the changes estimated by supply-side models were interpreted as physical output changes, which was critized as implausible by several authors [58, 59]. A reinterpretation of the supply-side model by Dietzenbacher [58] as a price model instead of a quantity model allowed for a meaningful use of the models. In this interpretation the quantities remain fixed and only the price of the outputs changes with a change in the inputs.

In this study the Ghosh-price model is used to estimate the impacts caused by a change of the capital stock resulting from a flood event on the outputs of an economy expressed as the change in prices.

In general, most IO case studies do not include uncertainty analyses [60]. Although, uncertainties due to e.g. errors in the collected data to the estimation of trade flows should not be ignored [61]. Also this study does not explicitly include uncertainties associated with the estimation of the indirect impacts, as the focus of this study has been more in the direction of linking the models including the uncertainties associated with estimation of the direct impacts. In theory the inclusion of uncertainties associated with the indirect impacts can be carried out, as suggested by Temurshoev [61], by e.g. perturbing the flow matrix similar to Bullard and Sebald [62]. This way, ranges of outputs reflecting possible variations of the trade flows can be obtained. However, this is only one possibility to approach this issue, many more such as the derivation of a probability density function of the Leontief inverse, are listed in Temurshoev [61].

### Linkage of direct and indirect economic impacts

Direct economic impacts of all federal states are aggregated for each economic sectors for whole Germany. Distributions of direct economic impact estimates for each of the 1000 different company versions, and therefore sector versions, are used to shock the IO model. Hence, the IO model is shocked 1000 times using 1000 direct economic impact distributions per economic sector in Table 1. Each direct economic impact distribution is represented by 1000 samples from the corresponding distribution. Subsequently, the approach described in the following is repeated 1000*1000 times for each economic sector covering all uncertainties associated with the direct economic impact estimation.

The calculated direct economic impact per sector is used as input to the Ghosh-price model to estimate the indirect economic impacts. The direct economic flood impact *D*_*j*_ is assumed to affect the capital stock of every economic sector *K*_*j*_ directly. The flood impacts on the capital stock of the companies are calculated as follows:

$$\begin{array}{c} K_{j}^{s}=K_{j}-D_{j} \end{array}$$ (3)

In a next step the value added *v* is recalculated by a summation of the labor *L* and the shocked capital stock *K*^*s*^:

$$\begin{array}{c} v^{s}=L+K^{s} \end{array}$$ (4)

The shocked value added *v*^*s*^ is then used to calculate the outputs *x*^*s*^ shocked by the flood event with:

$$\begin{array}{c} x^{s}={\left(I-B\right)}^{-1}v^{s} \end{array}$$ (5)

Since these results reflect the direct as well as the indirect economic impacts, a further differentiation between these two impacts is necessary. This is done by the subtraction of the direct from the indirect economic impacts before calculating the price changes.

$$\begin{array}{c} x_{j}^{s1}=\left(x_{j}-x_{j}^{s}\right)-D_{j} \end{array}$$ (6)

Consequently, the price change Δ*x* can be estimated as:

$$\begin{array}{c} \Delta x_{j}=x_{j}^{s1}/x_{j} \end{array}$$ (7)

In this case Δ*x*_*j*_ shows the decrease of output prices caused by an decrease of the inputs. This can be interpreted as the price decrease, which affected sectors have to compensate for.

## Results and discussion

First, the results of the identification of affected buildings and the estimates of the direct economic impact on companies of individual federal states as well as for the single economic sectors are presented and discussed. Second, the estimates of the indirect economic impacts and ratios between indirect and direct economic impact are shown.

### Direct economic impacts divided by federal states

**Fig 2:**
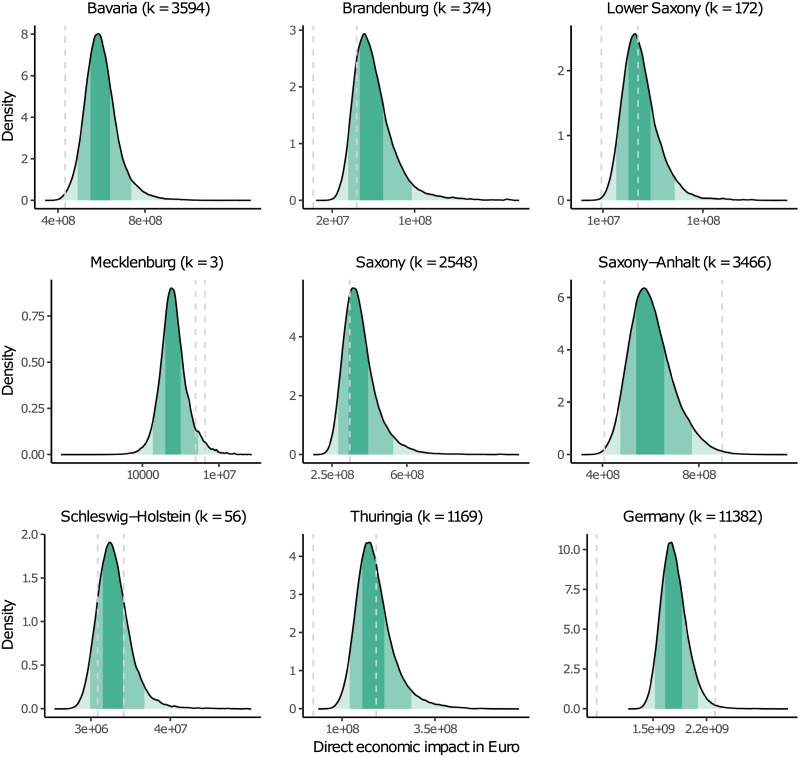
Distributions of estimated direct economic impact for k affected companies in the respective federal states. The grey dashed lines indicate the range of the assumed direct economic impact from reports of the respective federal states [44]. The different greens indicate the 50% and 90% intervals.

A total of 11382 companies affected by the flood in the eight federal states are identified by the overlap of the water mask with the OSM data (Fig 2). The Saxon Relief Bank, being responsible for the loss adjustment and management in Saxony after the flood in 2013, received about 2450 damage claims of companies. This number corresponds well with the number of 2548 affected companies identified in Saxony by means of the water mask and the OSM data (Fig 2). Comparable numbers for the other federal states are not available, but it is expected that this approach works equally well for all other federal states. Hence, in this case it can be assumed that the identification of flood affected buildings in Germany works reliably.

The overall direct economic impact to companies’ buildings and equipment is estimated per federal state (Fig 2). The distributions of the estimated direct economic impacts show the range of possible values. They reflect the uncertainties associated with the estimation of the direct economic impact. For example, the 50% interval (dark green) of the direct economic impacts suffered by companies in the federal state Saxony indicates an absolute direct economic impact between 300 and 380 million Euro, while the 90% interval (light green) indicates an absolute direct economic impact between 270 and 510 million Euro. Hence, according to these estimations there is a 90% probability that the direct economic impact lies between 270 and 510 million Euro. The higher the number of affected companies is in a federal state, the higher the corresponding direct economic impacts and the broader the ranges. The federal states Bavaria, Saxony-Anhalt and Saxony suffered the highest direct economic impact, whereby Schleswig-Holstein, Lower Saxony and Mecklenburg suffered the lowest direct economic impact. This is in accordance with the reported numbers published by the Federal Ministry of the Interior [63].

Generally, data for validation of estimated direct economic impact is scarce [2]. Therefore, reports of the Federal Ministry of Finance are used as benchmarks for a validity check [44]. These reports show mostly overall direct economic impacts per federal state including direct economic impacts to the sectors private households, industrial and commercial sector, agriculture and forestry as well as state and municipal infrastructure. Data about the share of the reported impacts by the different sectors is only available for the federal states Bavaria and Saxony. The share of the industrial and commercial sector to overall economic impacts ranges between about 15 and 35% [44]. Hence, this range was taken to check the validity of the estimated direct economic impacts to companies (gray dashed lines in Fig 2). For Bavaria the reported 32.4% of 1308 million Euro is taken as validity point and for Saxony the reported claims of companies amounting to about 306 million Euro to the Saxon Relief Bank is used. Note that these reported values are also prone to uncertainties and only give a rough idea about the direct economic impacts.

The validity check shows that ranges reported for each of the eight federal states lie within the distributions of estimated direct economic impact of the respective federal state. In some federal states, e.g. Bavaria and Brandenburg, the reported values are located towards the left tail of the distributions, suggesting an overestimation of the direct economic impacts. However, the derived percentages of 15 and 35% cover only the industrial and commercial sector, whereas the estimated direct economic impacts also cover service sectors such as education or arts, entertainment and recreation and partly infrastructure sectors such as electricity, gas, steam and air conditioning supply (Table 1). Therefore, the estimated impacts are expected to be higher than the values of the validity check, and we conclude that the distributions of the estimated direct economic impacts per federal state are plausible.

### Direct economic impacts divided by economic sectors

**Fig 3:**
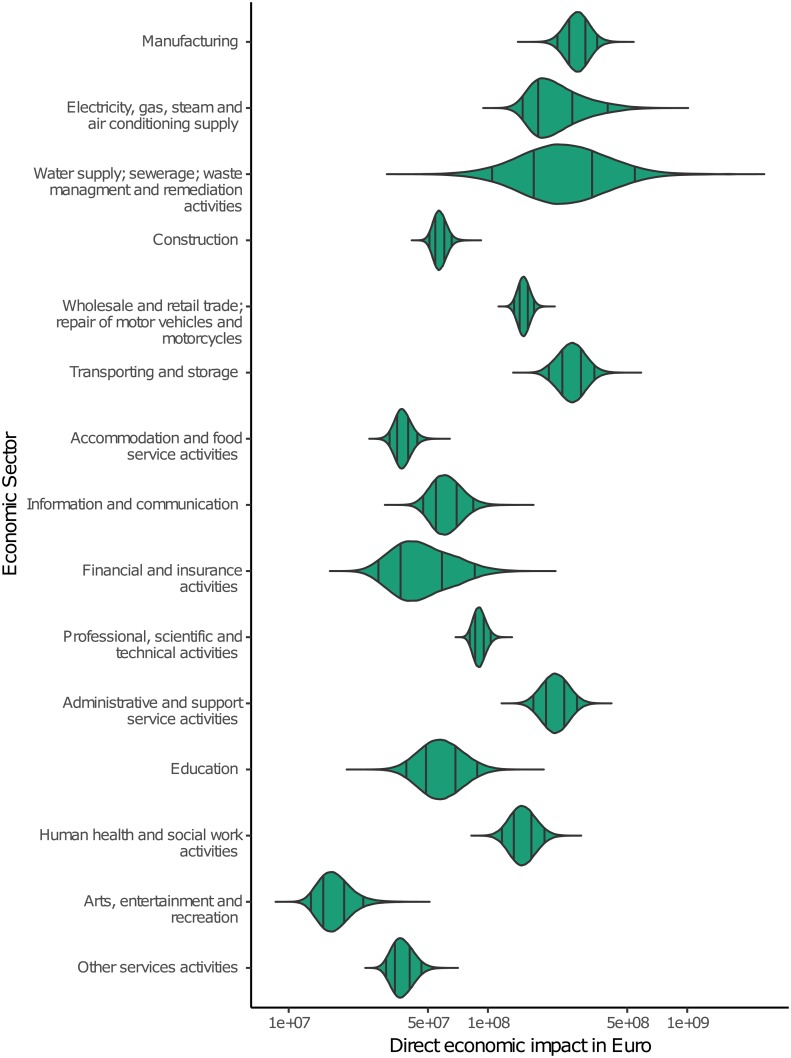
Distributions of estimated direct economic impacts per economic sector. The black lines indicate the 50% and 90% intervals.

The method of Sieg et al. [26] allows not only a spatial scaling (in this case to federal states), but also a thematic grouping, e.g. to economic sectors. Fig 3 shows the estimated direct economic impacts summed up to economic sectors over all eight federal states representing about 98% of the overall loss in Germany during the event. Data for a validity check with regard to direct impacts to the economic sectors for whole Germany was not available.

The distributions of the direct impacts vary widely between the sectors. The variation in the range is mainly caused by different asset values (Table 1) and company characteristics (Table 3). The difference in the location can be explained by the different asset values of the economic sectors and the number of companies of the respective sector in the federal states (Table 2).

Economic sectors which suffered the highest direct economic impacts are the Manufacturing, Electricity, gas, steam, and air conditioning supply, Water supply, sewerage, waste management and remediation activities, and Transportation and storage. These results seem plausible, since manufacturing companies are the most common and the asset values per employee of the other three economic sectors are high (Table 1). The lowest impacts are estimated for the sectors Arts, entertainment and recreation, as well as service activities which agrees with the comparatively low asset values of these sectors.

### Indirect economic impacts and ratio between indirect and direct impacts

**Fig 4:**
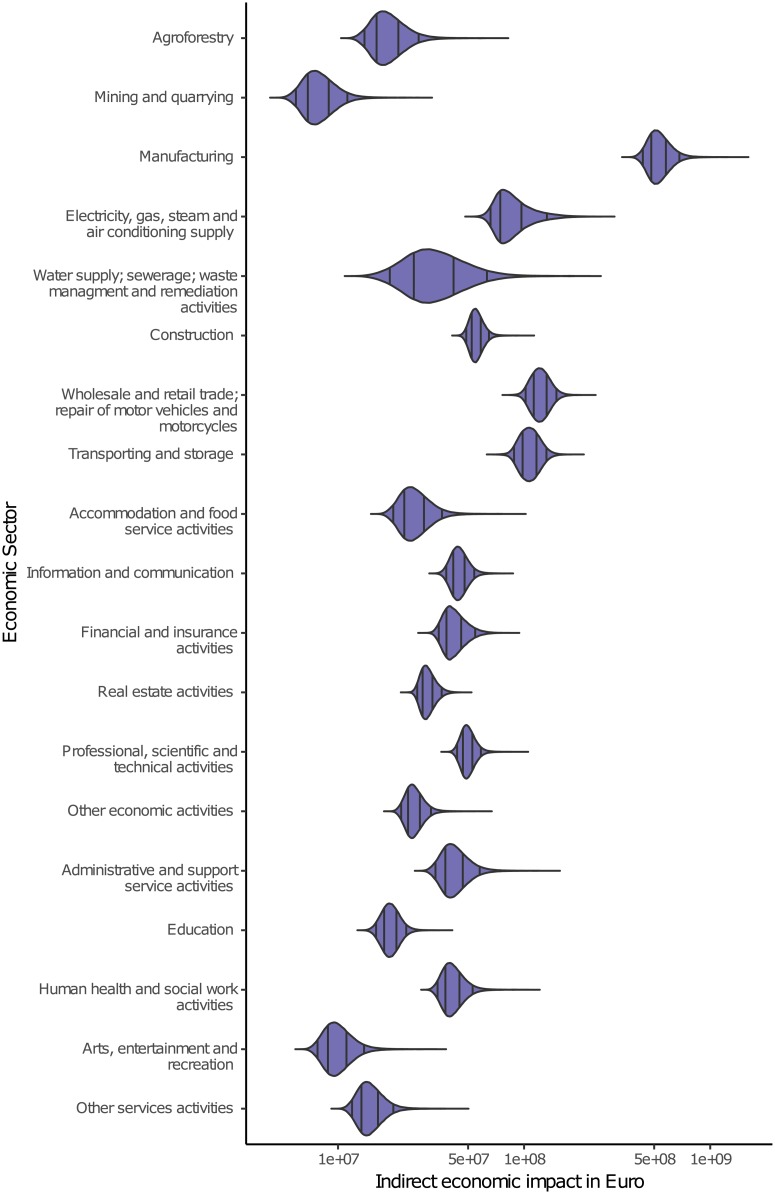
Distributions of estimated indirect economic impacts per economic sector. The black lines indicate the 50% and 90% intervals.

The indirect impacts, also sometimes named second-order or macroeconomic impacts, are induced by direct impacts which affect and permeate throughout the value chain. Examples are negative feedbacks to the wider economy, such as the production losses of suppliers and customers of the companies directly affected by the flood [7]. The distributions of the estimated indirect impacts vary correspondingly to the direct impacts (Fig 4). The distribution locations of the sectors Mining and qarrying, Arts, entertainment and recreation, and other service activities show the lowest indirect economic impacts, while Manufacturing shows by far the highest impact. Manufacturing companies are often particularly prone to indirect impacts due to their many dependencies on e.g. input factors (such as materials needed for production) or the supply chain [64, 65]. One example is reported during a major flood event in 2000 in Tokai, Japan, where many production processes in companies not directly hit by the flood were stopped due to disturbances in the supply chain and infrastructure damage [66]. Hence, the high indirect economic impacts suffered by the sector Manufacturing seem plausible. The low indirect impacts suffered by the sector Arts, entertainment and recreation can also be explained by to their relative independence from supply chains and inputs of other sectors.

**Fig 5:**
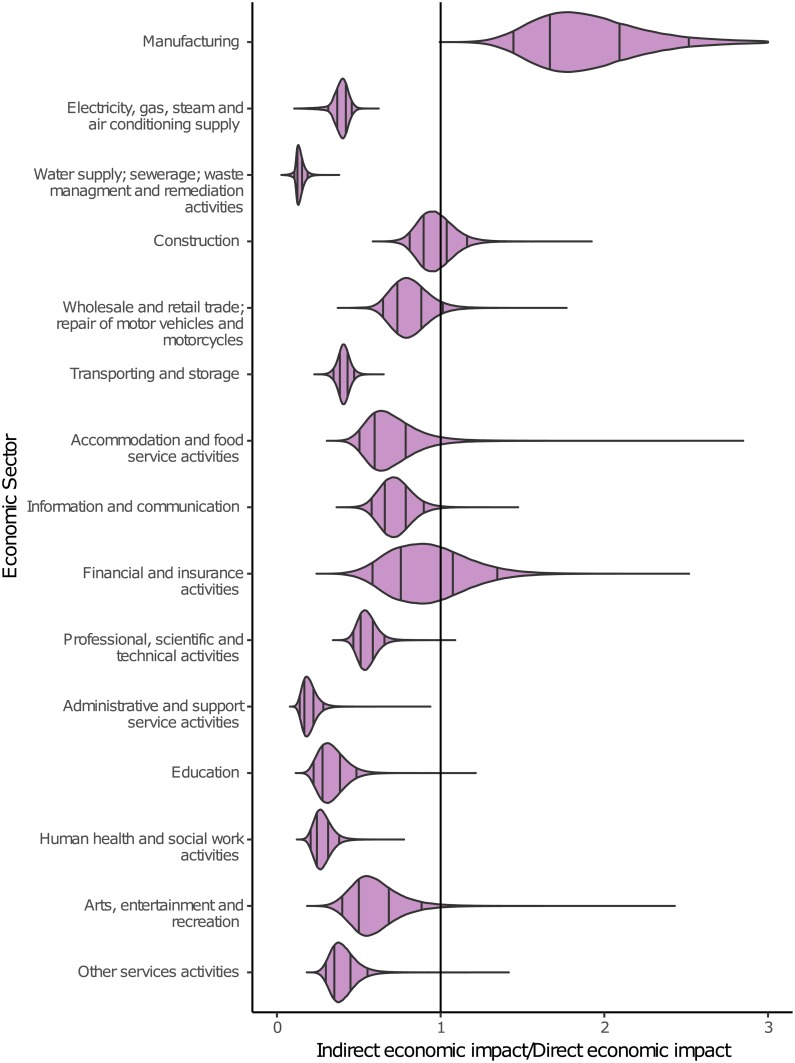
Distributions of the ratio between indirect and direct estimated economic impacts per economic sector. The black lines indicate the 50% and 90% intervals.

Fig 5 shows the ratio between indirect and direct impacts. The ratios show whether a specific sector suffers more from the direct or indirect economic impacts caused by a flood event. Again, the distributions vary strongly between the sectors. The sectors Water supply; sewerage; waste management and remediation activities, Administrative and support service activities and Human health and social work activities show the lowest ratios in the range of 0.11 and 0.26 considering the 90% intervals. Hence, direct economic impacts far exceed the indirect impacts on these sectors. The sector Manufacturing stands out as its complete distribution exceeds the value 1, indicating that this sector is mostly influenced by indirect impacts. Financial and insurance activities have a strong interrelation with other economic sectors and the economic growth [67]. Hence, although the direct economic impacts on this sector are rather moderate (Fig 3), the indirect impacts can be quite high explaining the rather high ratios (Fig 5). This variation in ratios between the economic sectors sheds doubt on the approach to use the direct impacts as proxy for indirect impacts. The distributions of the ratios represent variations of the flood event and the affected companies. Economic systems with a comparable economic and company structure are very likely to show ratios within the ranges of the distributions. Hence, these distributions of ratios are event unspecific and transferable between regions with similar economic systems.

**Fig 6:**
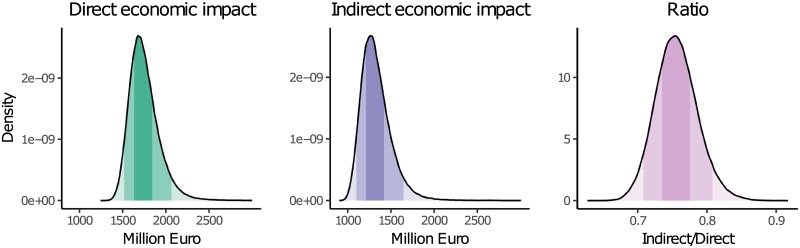
Distributions of estimated direct economic impacts (green), estimated indirect economic impacts (purple) and the ratio (pink) of both for Germany. The different shades of the colours indicate the 50% and 90% intervals.

Fig 6 shows the distributions of the direct and indirect economic impacts as well as the ratio accumulated for Germany. The direct economic impacts of the flood event 2013 in Germany lie with a probability of 90% between 1.5 and 2.1 billion Euro, while the indirect economic impacts lie between 1.1 and 1.6 billion Euro. The ratios for Germany range between 0.7 and 0.9 indicating that indirect impacts can almost be as high as direct impacts. This confirms the results of recent studies at the global scale, which observe direct and indirect economic impacts of floods to be almost even [68, 69]. In addition, these findings also support the point made by [32] that focusing on direct losses only, is insufficient for measuring disaster consequences.

Theoretically, the estimation of the indirect economic impacts can be applied at any spatial scale as the estimation of the direct economic impacts is scale independent. However, this is limited by the availability of Input-Output tables at the anticipated spatial scale. At the country level this information is readily available e.g. via the GTAP database [70]. These national IO tables could be further regionalized to lower spatial units, such as e.g. federal states under the use of substantial additional information for the anticipated scale (e.g. economic structures, regional GDP, employment data, commuting data, fiscal flows etc.). Hence, the transfer of this approach to other regions at the country level is more or less straightforward, while more effort is needed for the transfer to smaller administrative units.

## Conclusion

This study proposes a new modeling procedure for the joint assessment of direct and indirect economic flood impacts under uncertainties. For the first time an object-based estimation of direct economic flood impacts at the national level is successfully conducted. The resulting estimations are plausible compared to reported damage values. Hence, data sets containing individual buildings are suitable for the seamless estimation of direct economic flood impact at large spatial scales.

Within the same modeling procedure the direct economic impacts are linked to a supply-side IO model for estimating the indirect economic impacts. The ratio between indirect and direct economic impacts reveals to which kind of impact the economic sectors are more prone to. Large differences in this ratio between the economic sectors are identified. This indicates that the application of a single factor to direct economic impacts as a proxy for the indirect impacts is inappropriate. Furthermore, direct and indirect economic flood impacts are found to be almost equal, highlighting the importance to include indirect economic impacts in flood risk assessments and management.

The proposed procedure can be applied at any spatial scale, although a limiting factor is the data availability for IO models. This crucial data source is readily available at the country level allowing the application of the proposed approach at the country level in other regions as well, while an application to smaller spatial units is more challenging. The procedure’s probabilistic nature also allows its use for projections of the consequences of future flood events, since assumptions about possible future developments of e.g. the economy can be expressed by probability distributions. Therefore, uncertainties associated with the assumptions are captured.

Future research should investigate the uncertainty associated with the estimation of indirect economic impacts. CGE models could be integrated into the proposed procedure to analyze the long-term flood impacts. This model class allows for a more flexible economic adjustment process than IO models, which might better reflect real world circumstances in the medium to long-term. Furthermore, validating especially indirect economic impacts is an urgent challenge.

## Supporting information

S1 File Data sets used in Figs 2, 3, 4, 5 and 6. These files comprise values of the direct economic impacts per federal state (Fig 2) and values of the direct and indirect economic impacts per sector (Figs 3 and 4). The ratios can be computed with these values by dividing the indirect economic impacts by the direct economic impacts (Figs 5 and 6). The estimated economic impacts for whole Germany can be computed by adding up the the values in the different economic sectors (Fig 6).
(ZIP)
